# Physiology and pathophysiology of musculoskeletal aging: current research trends and future priorities

**DOI:** 10.3389/fphys.2013.00073

**Published:** 2013-04-09

**Authors:** Ali Mobasheri, Alexandrina F. Mendes

**Affiliations:** ^1^Faculty of Medicine and Health Sciences, Medical Research Council-Arthritis Research UK Centre for Musculoskeletal Ageing Research, Arthritis Research UK Pain Centre, Arthritis Research UK Centre for Sport, Exercise, and Osteoarthritis, The University of NottinghamNottingham, UK; ^2^Faculty of Pharmacy and Centre for Neuroscience and Cell Biology, University of CoimbraCoimbra, Portugal

“Physiology and Pathophysiology of Musculoskeletal Aging” is a Frontiers Research Topic aimed at increasing our understanding of healthy musculoskeletal aging. By gaining a better understanding of healthy musculoskeletal aging we can provide better care and new therapies for common musculoskeletal problems.

Sarcopenia is a term utilized to define the loss of muscle mass and strength and the consequent functional impairment that occurs with aging (Baumgartner et al., 1999; Waters et al., 2000; Morley et al., 2001). It can begin as early as the third decade of life. However, in most cases it affects individuals in the fourth decade and beyond. The gradual loss of muscle tissue (atrophy) reduces total body mass. Decreased physical activity with aging appears to be the key factor involved in producing sarcopenia (Morley et al., 2001). The age-related alterations in muscle involve age-related muscle fiber transitions and metabolic shifts in aging muscle. These alterations also affect posture and gait and become particularly evident in the seventh, eighth, and ninth decades of life. Mitchell and colleagues highlight these issues by reviewing the current knowledge of the decline in human muscle mass and strength with advancing age and the associated risk to health and survival (Mitchell et al., 2012). They also review the underlying changes in muscle characteristics and the etiology of sarcopenia. The authors draw on evidence from cross-sectional studies that have compared young and old muscle to show that in people aged 75 years, muscle mass is lost at a rate of 0.64–0.70% per year in women and 0.80–0.98% per year in men with more rapid concomitant losses in muscle strength; loss of muscle strength is thought to be 2–5 times greater than loss of mass. The loss of muscle strength is a more consistent risk factor for the development of disability and death than loss of muscle mass (Mitchell et al., 2012).

In their review article David Karasik and Miri Cohen-Zinder from Bar-Ilan University argue the need for identification of genes with pleiotropic functions in musculoskeletal aging (Karasik and Cohen-Zinder, 2012). The authors point out that musculoskeletal aging is detrimental to multiple bodily functions. This is a challenging area given the fact that the process of musculoskeletal aging becomes apparent in the fourth decade of an individual's life. Exploring pleiotropic relationships is difficult both methodologically and conceptually. However, in order to identify the biological mechanisms underlying these changes we need a “holistic” genetic approach to investigate the cross-talk between muscle and closely related tissues (i.e., tendon, bone, and cartilage). This strategy may allow us to find the links between skeletal muscle and other parts of the “musculoskeleton” (Karasik and Cohen-Zinder, 2012).

Kay Ohlendieck takes a molecular approach to musculoskeletal aging by reviewing the applications of proteomic profiling to aging muscle and the “fast-to-slow” muscle transitions that are thought to occur during aging (Ohlendieck, 2011a). The author has recently published a review article that discusses current proteomic approaches for studying skeletal muscle and associated technical challenges and emerging techniques (Ohlendieck, 2011b). His review article in this Research Topic discusses proteomic profiling approaches that have helped to establish an age-related shift to slower protein isoforms of myosin heavy chain, myosin light chain, actin and tropomyosin, as well as subunits of troponin (Ohlendieck, 2011a). These studies have confirmed previous assertions concerning the disproportionate age-related atrophy of type IIa muscle fibers and an age-related decrease in the synthesis rate of myosin heavy chain (Morley et al., 2001). Ohlendieck's mini-review also discusses the “glycolytic-to-oxidative” shift that occurs in slower-twitching senescent muscles and the newly identified proteins that are altered in aging muscle using proteomic profiling (Ohlendieck, 2011a). Proteomic profiling has also revealed an increase in mitochondrial enzymes and a concomitant decrease in glycolytic enzymes during the fast-to-slow transformation process in aging skeletal muscle. This timely mini-review which has previously been accompanied by a dedicated Editorial (Mobasheri, 2011b) also discusses alterations in metabolic and contractile elements that can be used to define a “sarcopenia-specific” biomarker signature.

Aging is also a major contributor to the development and progression of osteoarthritis (OA). OA is a long and slow continuum of joint changes and symptoms that has no clear-cut onset (Englund, 2010). Therefore, the use of “omics” technologies offers great promise for understanding factors that contribute to disease initiation and progression and how they are affected by aging.

Gharbi and co-workers (Gharbi et al., 2011) review the applications of proteomic techniques in OA research. One of the main objectives of applying proteomics to the study of cartilage aging and OA is to discover disease-specific biomarkers, which may reveal new therapeutic targets and facilitate targeted drug development. Gharbi et al., summarize proteomic techniques and their applications to OA research and discuss technical limitations and practical problems associated with sample preparation and strategies to overcome them. Proteomic studies of cartilage and other joint tissues will be particularly relevant to diagnostic and therapeutic research in OA (Mobasheri, 2011a).

Fernández-Moreno and colleagues from Blanco's group assess the link between a number of biomarkers and the mitochondrion-related phenotype in patients with OA (Fernandez-Moreno et al., 2012). They measured the serum levels of the OA-related biomarkers including matrix metalloproteinase-1 (MMP-1), matrix metalloproteinase-3 (MMP-3), matrix metalloproteinase-13 (MMP-13), myeloperoxidase (MPO), hyaluronic acid (HA), human cartilage glycoprotein 39 (YKL-40), cartilage oligomeric matrix protein (COMP), cathepsin K, and several peptides derived from type II collagen including Coll2-1, Coll2-1NO2, C2C, and CPII. The biomarkers were analyzed in 48 OA patients and 52 healthy controls carrying the mitochondrial haplogroups H and J. MMP-13 was the only biomarker that significantly increased in OA patients compared to healthy controls in both mitochondrial haplogroups H and J. The collagen type II biomarkers, Coll2-1, Coll2-1NO2, the Coll2-1NO2/Coll2-1 ratio, C2C, CPII, and the C2C:CPII ratio were significantly increased in OA patients carrying haplogroup H compared to OA carriers of the haplogroup J. Therefore, mitochondrial DNA haplogroups are potentially useful biomarkers of OA and may be used in conjunction with traditional protein biomarkers (Fernandez-Moreno et al., 2012).

Adrian Hunnisett and Christina Cunliffe put forward a positioning paper on the role of chiropractic treatment as a primary care intervention for better musculoskeletal health in the aging population in the United Kingdom (Hunnisett and Cunliffe, 2012). The elderly are encouraged to take more exercise to ensure, and maintain, musculoskeletal health. The authors propose chiropractic treatment as an Intervention in musculoskeletal aging and advocate patient-focused care for the management of musculoskeletal disorders. Chiropractic intervention and maintenance programs can promote health and ensure continued musculoskeletal functioning of the elderly population and encourage the mechanistic and evidence-based integration of chiropractic into the National Health Service of the United Kingdom (Hunnisett and Cunliffe, 2012).

Musculoskeletal aging is a complex process. It involves tissue atrophy and loss of function in muscle, bone, tendon, ligament, intervertebral disk, and articular cartilage. Musculoskeletal aging is also accompanied by reduced neuromuscular integrity. The progressive loss of musculoskeletal mass and function highlights the importance of physical activity and exercise in elderly people. Increased adiposity (sarcopenic obesity) is another mechanism that contributes to age-related musculoskeletal deterioration. Increasing exercise capacity and physical fitness are likely to results in regeneration and remodeling within musculoskeletal tissue compartments. However, the molecular mechanisms that underscore these changes are poorly understood. Understanding age-related musculoskeletal deterioration requires co-ordinated basic, clinical, and translational studies that can generate novel and clinically testable approaches to healthier musculoskeletal aging. The elegant and original articles in this Research Topic highlight some of the major issues facing researchers in the area of musculoskeletal health and aging. We hope that this Research Topic will serve as a platform for encouraging further research into musculoskeletal aging.
